# The two faces of DNA oxidation in genomic and functional mosaicism during aging in human neurons

**DOI:** 10.3389/fragi.2022.991460

**Published:** 2022-10-12

**Authors:** Michael A. Lodato, Jennifer S. Ziegenfuss

**Affiliations:** University of Massachusetts Chan Medical School, Worcester, MA, United States

**Keywords:** somatic mutation, oxidative damage, neurodegeneration, aging, epigenetics, mutation signature, single-cell ‘omics, 8-oxo-2’-deoxoguanosine

## Abstract

Maintaining genomic integrity in post-mitotic neurons in the human brain is paramount because these cells must survive for an individual’s entire lifespan. Due to life-long synaptic plasticity and electrochemical transmission between cells, the brain engages in an exceptionally high level of mitochondrial metabolic activity. This activity results in the generation of reactive oxygen species with 8-oxo-7,8-dihydroguanine (8-oxoG) being one of the most prevalent oxidation products in the cell. 8-oxoG is important for the maintenance and transfer of genetic information into proper gene expression: a low basal level of 8-oxoG plays an important role in epigenetic modulation of neurodevelopment and synaptic plasticity, while a dysregulated increase in 8-oxoG damages the genome leading to somatic mutations and transcription errors. The slow yet persistent accumulation of DNA damage in the background of increasing cellular 8-oxoG is associated with normal aging as well as neurological disorders such as Alzheimer’s disease and Parkinson’s disease. This review explores the current understanding of how 8-oxoG plays a role in brain function and genomic instability, highlighting new methods being used to advance pathological hallmarks that differentiate normal healthy aging and neurodegenerative disease.

## Introduction

Aging is a multi-modal complex process that leads to the loss of physiological functions resulting in an increased risk of disease and death (Childs et al., 2015). Aging is the most critical risk factor among sporadic neurodegenerative disorders such as Alzheimer’s (AD) and Parkinson’s disease (PD). One of the many cellular factors hypothesized to contribute to age-induced neurological defects is damage to DNA, which is hazardous because DNA is the blueprint for all cellular functions. The idea that mutations in an organism can negatively affect fitness was proposed as early as the 1930s (Haldane, 1937). Since then, several factors have been found to contribute to DNA mutational damage, including radiation, environmental carcinogens, spontaneous deamination due to the inherent instability of nucleic acids, and oxidative products of metabolism (Lindahl, 1993). In fact, the human genome accumulates up to an estimated 120k lesions per day (Bauer et al., 2015). Persistent DNA damage can trigger genotoxic stress signaling that drives cell death. To combat this daily genomic threat, the cell uses highly conserved mechanisms to detect DNA damage and damaging agents to repair DNA lesions before they become permanent mutations.

The long-term maintenance of genomic integrity is paramount for proper homeostasis of all cellular functions, including in energetically expensive neurons. Neurons are some of the most metabolically demanding cells in our body, as they consume ∼80% of the energy used in the brain to support their signaling activity (Hyder et al., 2013). Age-associated damage is particularly dangerous in neurons because they are post-mitotic and unable to be replaced but must survive throughout the lifetime of an organism. Energy requirements are increased in brain regions dependent on increased neuronal activity, such as areas with high synaptic connections (Harris et al., 2012). Thus, neurons display high oxidative metabolic demands, so they are at increased risk of substantial oxidative damage. Oxidative damage to a cell has long been linked to aging by the free radical theory of aging proposed by Denham Harman in the 1950s (Harman, 1956), which posits that age-dependent accumulation of oxidative damage to cellular macromolecules causes a progressive deterioration of cells, tissues, and organs necessary for the function of the organism.

## Cellular and molecular responses to a heightened oxidative state

DNA experiences damage during an individual’s lifetime due to hydrolysis, encounters with reactive metabolites, or environmental chemicals. Human neurons will lose ∼10^8^ purines, or ∼3% of their total purine residues from their DNA, due to heat-induced frequent depurination, in an individual’s lifetime (Lindahl and Nyberg, 1972). Pyrimidines are more resistant to these forces but are nevertheless also attacked by free radicals. The human body produces oxygen free radicals and other ROS as by-products of numerous physiological and biochemical processes. In mitochondria, electrons can leak from the respiratory chain and generate ROS, while ROS can also be generated from exposure to ionizing radiation, chemicals, environmental factors, or immune functions (Ames et al., 1993). The imbalance between ROS’s cellular production and cells’ ability to efficiently defend against it is called oxidative stress. Oxidative stress has been specifically implicated in age-related cognitive decline and pathophysiology of many neurodegenerative diseases, particularly AD and PD (Jenner and Olanow, 1996; Simonian and Coyle, 1996; Markesbery, 1997; Mariani et al., 2005; Guo et al., 2018). It can cause cellular damage and subsequent cell death because the ROS oxidizes vital cellular components such as lipids, proteins, and DNA. The brain is exposed throughout life to excitatory neurochemicals which induce the activity of mitochondria, whose metabolism is like a busy factory producing constant oxygen-related free radicals like superoxide and hydroxyl radicals, or reactive species such as hydrogen peroxide and nitric oxide (Halliwell, 1994). Indeed, during the protracted neuronal activation associated with epilepsy, cellular macromolecules, including DNA, show elevated levels of oxidative damage (Bruce and Baudry, 1995) (Jarrett et al., 2008). Thus, neurons are in a tenuous position of requiring long-term survival while experiencing intense oxidative damage pressure to their genomes due to their functionality in biological circuits. This accumulation of genotoxic stressors can result in age-related cellular senescence, functional deficits, and unique perturbations that correspond with degenerative disease.

The free radical theory of aging shares commonalities with the somatic mutational theory of aging; ROS can act as a mutational source generating lesions that can ultimately result in permanent somatic mutations accumulating in the genome. Somatic mutations originate from exogenous and endogenous mutational processes in a cell and the cells’ lineage after fertilization. These mutations may be benign, but they can also potentially represent a slow and malevolent force inducing permanent deleterious changes to the genome. It has only recently become possible to accurately study somatic mutations in normal and pathologic tissues thanks to the development of new sequencing technologies such as single-cell and duplex sequencing, along with the development of accurate algorithms to identify mutations in these data (Schmitt et al., 2012; McConnell et al., 2013; Cai et al., 2015; Evrony et al., 2015; Hoang et al., 2016; Knouse et al., 2016; Dong et al., 2017; Bohrson et al., 2019; Perez-Rodriguez et al., 2019; Abascal et al., 2021; Gonzalez-Pena et al., 2021; Luquette et al., 2021). Studies using these new technologies have identified “mutational signatures,” comprising specific classes of mutations resulting from the activity of various DNA damage agents and repair pathways. More than 150 mutational signatures are currently identified in humans (Petljak et al., 2019; Alexandrov et al., 2020; Degasperi et al., 2022). Like finding fingerprints at the scene of a crime, the analysis of the activity of somatic mutational signatures has been used to provide insight into underlying causes and potential therapies for biological dysfunction, such as cancer (Seow et al., 2016; Chuk et al., 2017).

Since somatic mutations are known to increase cancer risk, as each mutation has the chance of being oncogenic (Failla, 1958), could somatic mutations contribute to a shorter lifespan, age-related functional deficits, and age-related diseases? Recently, whole genome sequencing from various species with a range of sizes and lifespans found that all samples showed an age-associated accumulation of somatic mutations and signatures, including oxidative damage. With increasing age, humans show a steady increase in genomic somatic mutation burden and rate, as well as mutational signatures, which indicate a heightened level of oxidative damage (Lodato et al., 2018; Zhang et al., 2019; Brazhnik et al., 2020). These data support the hypothesis that mutational load contributes to diseases of aging and ultimately resides at the crux of limiting human lifespan (Failla, 1958; Szilard, 1959).

## The negative face of oxidative changes to the genome: role of 8-oxoG in genotoxicity and aging

**FIGURE 1:**
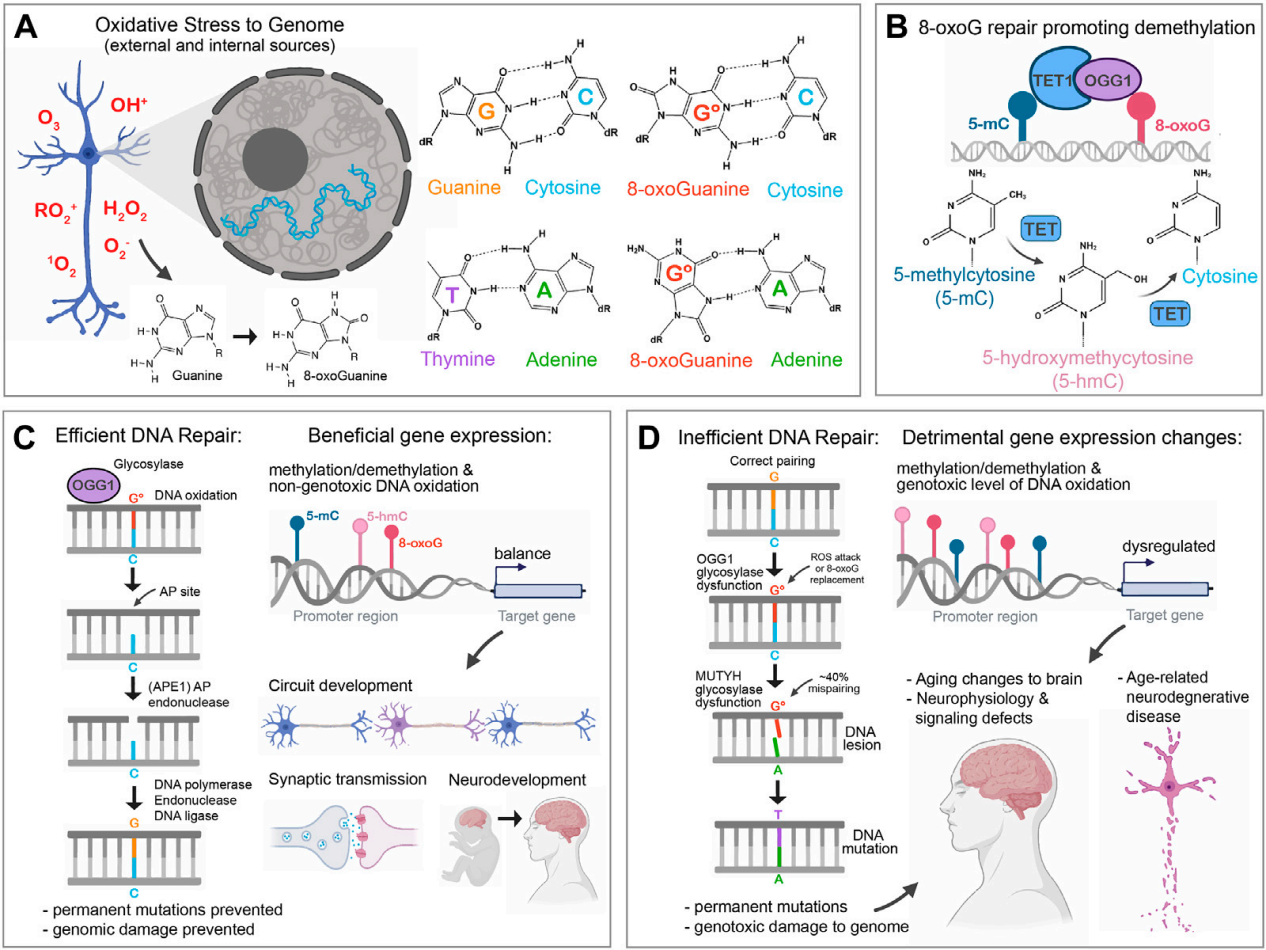
The beneficial and detrimental sides of 8-oxoG on the genome and gene expression. **(A)**. Oxidative stress molecules can change nucleotides by adding oxygen to the molecular structure, such as turning guanine into 8-oxoguanine (8-oxoG). This can occur to free nucleotides, which later intercalate into DNA, or via direct attack of guanosine in DNA. 8-oxoG can easily pair with cytosine, which is indistinguishable from a normal guanine-cytosine pairing. 8-oxo prefers to pair with cytosine, but sterically can form a Hoogsteen base pair with adenine. An 8-oxoG-adenine mispairing is unstable, with adenine preferring to pair with thymine. **(B)**. The presence of 8-oxoG can indirectly promote the demethylation of nearby methylated cytosine (5-mC), via OGG1. OGG1 is the glycosylase in charge of removing 8-oxoG from DNA, but can also partner with TET1, and enhance its enzymatic activity as promoting 5-mC demethylation into forms such as 5-hmC which can promote gene expression changes via epigenetic mechanisms. **(C)**. In a well-regulated system, the presence of 8-oxoG in the genome is efficiently removed from the DNA via the Base Excision Repair pathway. Once 8-oxoG is excised from the DNA, molecules such as APE1 continue to fix the resulting AP site by nicking the phosphate backbone and initiating polymerase-based repair. Thus, the genome is protected from permanent somatic mutational damage. 8-oxoG in such a robust repair environment is limited to having a temporary presence in DNA, which can potentially promote brief changes in OGG1- > TET methylation changes and activation in gene promoters. This can promote the gene expression changes found to be essential in many neurodevelopmental events, such as gross brain development, neuron maturation, circuit development, and synaptic function. It should be noted that in addition to BER, the NER proteins, can play a role in clearing 8-oxoG using glycosylase enzymes like OGG1 (Kumar et al., 2022). **(D)**. In a cellular environment with excess oxidative stress, increased 8-oxoG can overwhelm repair machinery. The longer perdurance of 8-oxoG can lead to a greater chance at mispairing with adenine at a rate of ∼30%. This 8-oxoG-adenine lesion can result in the establishment of a permanent somatic mutation in the DNA. This genotoxic mutational damage can accumulate over time and lead to detrimental changes in the brain. An excess of 8-oxoG can potentially also affect OGG1 and TET-mediated cytosine methylation/demethylation balance, which results in dysregulated gene expression. Dysregulated gene expression in concert with accumulating somatic mutations to the genome is associated with age-related changes to the brain, including neuronal function and neurodegenerative disease.

ROS inflicts oxidative modifications on various biological molecules, and one of the most common genotoxic stressors to DNA is 8-oxo-7,8-dihydroguanine (8-oxoG). 8-oxoG in the genome can originate from the nucleotide pool being oxidized, which can incorporate into nuclear or mitochondrial DNA during replication or repair, or via direct oxidation of the DNA guanosine base (Kasai and Nishimura, 1984; Nakabeppu et al., 2006). The potent mutagenicity of 8-oxoG is due to its ability to sometimes evade detection by DNA damage polymerases by adopting Hoogsteen base pairing with adenine in a semi-stable Watson-Crick geometry. As a consequence of mispairing with adenine during DNA synthesis, 8-oxoG can cause G:C > T:A transversions (i.e., C > A mutations) (Figure 1A). *In vivo* steady-state kinetics or biophysical transition path sampling experiments have shown that 8-oxodGTP is still preferentially paired (2:1) with dCTP over dATP in DNA, but the potential for a mispair still occurs at a rate of ∼30% (Miller et al., 2000; Wang and Schlick, 2007). 8-oxoG and its genotoxic consequences are normally efficiently mitigated by the following DNA-damage repair (DDR) enzymes, 1) 8-Oxo-7,8-Dihydroguanosine Triphosphatase (MTH1/NUDT1) which hydrolyzes oxidized free purine nucleotides, 2) the base excision repair (BER) protein adenine DNA glycosylase (MUTYH/MYH) which removes adenines misincorporated opposite to 8-oxoG, and 3) the BER protein 8-oxoguanine DNA glycosylase (OGG1) which is the only factor that can directly excise 8-oxoG incorporated in DNA (Michaels et al., 1992; Grollman and Moriya, 1993). OGG1-mediated excision of the 8-oxoG base from DNA leaves an apurinic/apyrimidinic (AP) site, the substrate for apurinic/apyrimidinic endonuclease 1 (APE1). APE1 hydrolyzes the phosphodiester bond on the AP site resulting in a single-strand break, which is finally repaired by DNA polymerases and DNA ligase (Figure 1C). In addition, the nucleotide-excision repair (NER) proteins XPA, XPC, XPG, CSA, CSB, and UV-DDB can stimulate the activity of the BER pathways involved in repairing 8-oxoG (Kumar et al., 2020). Furthermore, when excision and repair of 8-oxoG by OGG1/APE1 leads to a transcription block by stalling Pol II, XPA is recruited as part of a transcription-coupled nucleotide-excision-repair (TC-NER) pathway to alleviate the block (Kumar et al., 2022). Interestingly, 8-oxoG repair is severely inhibited by the proximity to other kinds of DNA damage (such as mismatches, unfixed abasic sites, and single-strand breaks) (David-Cordonnier et al., 2001); therefore, an abundance of glycosylase and endonuclease-mediated 8-oxoG repair factors could negatively affect future repair. If this genotoxic assault is not accurately and quickly repaired, oxidative damage via mutagenic agents like 8-oxoG will accumulate in DNA, which has been associated with various aging processes such as senescence and disease (Ames, 1983; Finkel and Holbrook, 2000) (Figure 1D).

Several lines of evidence suggest that cellular oxidative damage correlates with aging and neurodegenerative disease. High amounts of oxidized proteins in the cortex correlate with age-related deficits in spatial memory in mice (Forster et al., 1996). In the human brain, HPLC analysis showed a progressive age-related accumulation of 8-oxoG in DNA in both the cerebellum and cortex (Mecocci et al., 1993). A similar HPLC-based analysis in multiple cortex regions in AD and neurotypical brains showed an increase in 8-oxoG in both nuclear and mitochondrial DNA compared with age-matched controls (Mecocci et al., 1994). Levels of 8-oxoG were also significantly higher in peripheral blood DNA of AD patients relative to controls as assayed by ELISA (Sliwinska et al., 2016). 8-oxoG immunoreactivity was present in 35% of substantia nigra (SN) neurons from PD patients with dementia, while less than 10% of SN neurons from age-matched neurotypical patients showed measurable 8-oxoG (Zhang et al., 1999). These early studies showed that cellular oxidation is correlated with neuron vulnerability from a degenerative disease.

In response to increased cellular oxidation, DNA repair enzyme activity increases during aging and in neurodegenerative disease. The expression of the repair enzymes for 8-oxoG (OGG1, MUTYH, and MTH1) increase in the mitochondria and cytoplasm of SN neurons from sporadic PD patients (Shimura-Miura et al., 1999; Fukae et al., 2005; Arai et al., 2006), suggesting that cells experiencing high 8-oxoG also express high levels of DNA damage repair enzymes. MTH1 increases in the entorhinal cortex of the hippocampus, a focal point of AD, in sporadic AD patient brain (Furuta et al., 2001). OGG1 immunoreactivity corresponding to the cytoplasmic/mitochondrial compartment of the cell was is lower in AD than in age-matched control brains, but does co-locate with Tau tangles within the cytoplasm (Iida et al., 2002). These observations indicate that there is a reduced capacity of OGG1 and/or MTH1 to repair damage to nucleotides, which may account for specific types of accumulated AD-related 8-oxoG oxidative damage. MUTYH increases in hippocampal neurons and glial cells from AD patient brains (Mizuno et al., 2021). In MUTYH knock-out mice, neurodegeneration, microgliosis, and tumorigenesis linked to 8-oxoG accumulation are suppressed (Oka and Nakabeppu, 2011; Sheng et al., 2012). Upon further investigation in a mouse model of AD, MUTYH deficiency was found to improve hippocampal neurogenesis, improve behavioral and cognitive impairments, and significantly decrease microgliosis (Mizuno et al., 2021). Thus, oxidative damage inducing a MUTYH-mediated repair process may contribute to patient memory impairment in an early phase of AD as well as pathogenesis through excessive microglial activation.

8-oxoG shapes the aging and AD neuronal genome and could act as a biological read-out of genotoxic stress and somatic mutation. The abundance of 8-oxoG in neurotypical brain samples increases significantly with advanced age compared to young adults (Miller et al., 2022). Interestingly, this mirrors the single-cell somatic mutational analysis of single neurons in young versus aged brains, where a significant age-related increase in somatic mutations occurs (Lodato et al., 2018). Single neuron somatic mutations comprise two distinct Signatures, named Signature A and Signature C (Lodato et al., 2018). Signature C features C > A substitutions that can be induced by 8-oxoG, and suggesting that advanced age-related somatic mutations occur downstream of ROS. 8-oxoG is also increased in the brains of advanced AD patients compared to neurotypical age-matched patient brains (Miller et al., 2022). Notably, Signature C closely corresponds to COSMIC signature SBS8, which is associated with brain cancer and defects in DNA repair of oxidized guanine (Alexandrov et al., 2020). Single excitatory neurons from advanced AD cases show a significant increase of somatic mutations throughout the genome compared to neurotypical age-matched neurons, with Signature C particularly prevalent in AD versus normal cells (Miller et al., 2022). This finding demonstrates that oxidative damage to guanine nucleotides is not only found in “normal” aging but also exceptionally predominant in idiopathic AD. Of note, somatic mutations in general and Signature C in particular increase in early-onset neurodegenerative disorders originating from NER enzymes (Lodato et al., 2018). Both Signature A and C contain C > T mutations at CpG dinucleotides, and 8-oxoG is enriched at CpG sites (see below), suggesting that 8-oxoG may also play an indirect role in C > T mutations comprising both signatures.

Taken together, this collection of research shows that 8-oxoG is increased in areas that mirror the main neurodegenerative and pathologic brain regions and cell types in both AD and PD. Genomic analysis along with *in vivo* cellular analysis connect oxidative status with the degree of genomic damage that may adversely impact genomic stability.

Several lines of evidence suggest that somatic mutations directly cause cellular dysfunction, particularly in the brain. DNA repair diseases such as Cockayne Syndrome (CS; caused by defective TC-NER), Xeroderma Pigmentosum (XP; defective global NER), and Ataxia telangiectasia (defective double-strand break repair) result in progressive neurodegeneration of specific classes of neurons (Lehmann, 2003; Hoche et al., 2012), suggesting that neurons rely on genome integrity to maintain their survival. Data from CS/XP mouse models and human patients suggest that unrepaired DNA damage accumulates in these diseases and causes cell death *via* transcriptional dysregulation (de Vries et al., 1997; Giese et al., 1999; Dolle et al., 2006; Tiwari et al., 2021), and our work showed that somatic mutations also increase in human CS and XP patient neurons (Lodato et al., 2018). Outside of the brain, somatic mutations 1) induce transcriptional instability in the human pancreas during aging (Enge et al., 2017), causing insulin-secreting β-cells to secrete the insulin antagonist glucagon, and 2) decrease self-renewal potential in muscle stem cells during human aging (Franco et al., 2018). Our previous work suggests that when mutations randomly accumulate throughout the genome of a single cell, the chance of two deleterious independent mutations occurring on two alleles of the same gene rises exponentially during human aging (Lodato et al., 2018) and in AD (Miller et al., 2022). Nonsynonymous mutations that do not alter gene function can still create neoantigens, resulting in the recruitment of cytotoxic T-cells to sites of high mutation burden. Indeed, cytotoxic T-cells invade the human (Gate et al., 2020) and mouse (Groh et al., 2021) brains during neurodegeneration. Across mammals, species with long lifespans accumulate somatic mutations more slowly than short-lived species (Cagan et al., 2022), linking genome maintenance and longevity. Thus, while the exact mechanisms by which DNA damage and somatic mutations impact neurodegeneration remain undefined, one or more of these mechanisms may play a vital role.

## The positive face of oxidative changes to the genome: 8-oxoG in the regulation of neuronal gene expression

The evidence that DNA oxidation and repair are involved in aging and disease suggests that it is simply an accidental by-product of aerobic metabolism and triggers undesirable genotoxic damage. However, a lower level of “physiological” DNA oxidation and repair activity may play an important role in developmental and cognitive fitness. Recent evidence has supported the idea that a regulatory function exists for 8-oxoG lesions in gene promoters. The presence of 8-oxoG in its promoter increases VEGF gene expression (Pastukh et al., 2015; Fleming et al., 2017), promoter-associated OGG1 and 8-oxoG are coupled with increased NF-kB expression (Pan et al., 2016), and RNA knockdown of OGG1 impairs neuronal stem cell differentiation and decreases gene expression of both neuronal and astrocytic genes (Reis and Hermanson, 2012). Additionally, 8-oxoG forms a complex with OGG1 acts as a guanine exchange factor (GEF) in the cytoplasm in response to oxidative stress (Boldogh et al., 2012), which then helps promote signaling pathways which leads to transcription of genes involved in stress response defenses (Radak and Boldogh, 2010; German et al., 2013; Hajas et al., 2013; Aguilera-Aguirre et al., 2015). These observations strongly suggest that 8-oxoG integration and repair plays a role in development, survival, and normal tissue homeostasis.

The genomic distribution of 8-oxoG is not uniform in DNA, with high cell-to-cell variability of 8-oxoG across a population of cells (Ohno et al., 2006; Yoshihara et al., 2014). Recently, the sequencing of immunoprecipitated 8-oxodG-containing fragments using OxiDIP-Seq demonstrated that 8-oxoG incorporated within promoter regions and gene body of transcribed long genes in mammalian cells (Amente et al., 2019). This result is in line with high-resolution 8-oxodG sequencing (OG-Seq), that labeled 8-oxoG with biotin for affinity purification prior to next-gen sequencing, and showed distribution of 8-oxoG in mouse embryonic fibroblasts was lower in intergenic regions compared to genic regions (Ding et al., 2017). These data may reflect the relative C-G-rich nature of coding and regulatory regions relative to non-coding regions (Louie et al., 2003; Amit et al., 2012). Interestingly, the preferential accumulation of 8-oxoG in regulatory hotspots, like C-G-rich promoters, 5′-UTRs, and 3′-UTRs in the genome suggests an increased likelihood that transcriptionally active euchromatic regions are being targeted by guanosine oxidation and that 8-oxoG can play a role in the regulation of genomic functions.

Classically, epigenetic regulation is associated with the methylation and demethylation of cytidine in DNA, with 5-methylcytosine (5-mC) nucleotides playing important roles in regulating gene expression at promoters, gene bodies, and enhancers. Yet, 8-oxoG has also been shown to negatively affect active cytosine methylation by interfering with the binding of DNA methyltransferases to cytosine (Weitzman et al., 1994; Maltseva et al., 2009). Additionally, neighboring 5-mC nucleotides flanking 8-oxoG lesions show a reduced affinity for methyl-CpG binding proteins, which reduces transcription suppression efficiency of 5-mC and suggests that 8-oxoG exerts a long-lasting effect on gene expression (Valinluck et al., 2004). These studies extend the epigenetic landscape in DNA beyond methylation to include oxidation of specific nucleotides, like 8-oxoG, which can modify gene expression that helps the cell and organism.

Active DNA demethylation is an important part of transcriptional regulation in neurons to fine-tune cell physiology for neuronal plasticity and cognitive function (Li et al., 2013). 8-oxoG affects the methylation state of nearby 5-mC (Weitzman et al., 1994; Turk et al., 1995) indirectly through OGG1(Zhou et al., 2016). OGG1 binding to 8-oxoG recruits TET1, which is an active DNA demethylase (Tahiliani et al., 2009), and this DNA oxidation directly promotes DNA demethylation by converting nearby 5-mC sites to 5-hydroxymethylcytosine (5-hmC) (Zhou et al., 2016) which is thought to play crucial roles in gene expression regulation (Figure 1B). Indeed, 5-hmC has been shown to increase during neurodevelopment, especially around pro-neurogenesis gene regions (Colquitt et al., 2013; Perera et al., 2015). TET enzymes, along with 5-hmC, were also found to be essential during cerebellar circuit formation (Zhu et al., 2016), hippocampal neurogenesis (Zhang et al., 2013), and even in the regulation of synaptic transmission and plasticity (Yu et al., 2015). The correlation between 8-oxoG repair, TET enzymes, and 5-mC demethylation suggests a model where oxidative stress recruits OGG1/TET1 complex proteins to 8-oxoG and facilitates 5-mC conversion to 5-hmC and subsequent important changes to gene expression essential for development (Figures 1B,C).

Neurons are highly metabolic and developmentally and functionally complex, requiring dynamic regulation of the transcriptome during development and in the adult. 8-oxoG production increases when energy demand is high, potentially bridging metabolic changes to the changing transcriptional demands neurons experience throughout life. This relationship between metabolism and epigenetics has also been observed in embryonic stem cells (Wu et al., 2011). By being incorporated near gene promoters, 8-oxoG may act as another layer of regulation and help establish a transcriptionally permissive chromatin environment surrounding activated neuronal genes in both developing and adult brains in response to lived experience. Of particular note, CpG methylation can be mutagenic with 5-mC being approximately five-fold more likely to undergo deamination and resulting in a C > T mutation (Ehrlich et al., 1986). This particular mutation arising from deamination is represented as COSMIC signature SBS1, which is clock-like in that it steadily increases with age in all cells throughout the body, including neurons. It is currently unclear how a OGG1/TET1 complex recruited to 8-oxoG affects cytosine methylation over the course of aging and how potential changes in efficacy may factor into the SBS1 signature.

## Antagonistic pleiotropy: reconciling beneficial and deleterious genomic effects of 8-oxoG

Here we discussed the various detrimental effects of 8-oxoG on aging and in neurodegenerative diseases, but how do we reconcile the well-described negative effects of oxidative damage and increased somatic mutational burden with the role of 8-oxoG acting as an epigenetic regulator of gene expression in healthy cells? In 1957, George Williams postulated the existence of pleiotropic genes having opposite effects on fitness at different ages of an organism, such that the genetic effects were beneficial in early life, when natural selection is strong, but harmful at later ages, when selection is weak (Williams, 2001). This idea is now known as the “antagonistic pleiotropy” theory. While 8-oxoG is not itself a gene, it is a product of a cells’ physiological state and can act as a modulator of genetic expression. Although 8-oxoG always has the potential for destabilizing the genome, the level of 8-oxoG seemingly is kept low enough via antioxidants and repair mechanisms to prevent permanent somatic mutations through the majority of the organism’s lifespan (Figure 1C).

Later in life, dysregulated redox reactions and higher ROS lead to higher 8-oxoG abundance that induces a higher rate of permanent mutations. An unintended side effect of this genotoxic stress is potentially the upregulation of abnormal gene expression through 8-oxoG’s role in DNA demethylation (Figure 1D). Indeed, epigenetic analyses using iPSCs from neurotypical and AD patient neurons showed differential cytosine hydroxymethylation in genes associated with AD susceptibility and synaptic function (Fetahu et al., 2019). A genome-wide analysis also revealed a specific reduction of 5-hmC in neurons from a transgenic AD mouse model (Zhang et al., 2020). DNA methylation and hydroxymethylation gene dysregulation has also been implicated in PD, as TET1 mutations were reported in some PD patients (Shu et al., 2019). Loss-of-function mutations in TET enzymes were found in early-onset AD patients (Cochran et al., 2020), along with the observation that 5-hmC abundance and TET enzymatic activities decreased in AD patient brains (Zhang et al., 2020), suggests that TET reduction is the main cause for the loss of 5 hmC in disease contexts. These observations warrant further studies to clarify how roles of 8-oxoG and OGG1 change with aging and how their dysregulation may affect changes to signaling partners that can change epigenetic methylation status within the neuron in events leading to neurodegenerative disease onset.

This late-life deleterious side effect of a process that is beneficial in earlier life is of little consequence for natural selection, thus 8-oxoG effects during early-life versus late-life may be an example of the underlying principle of antagonistic pleiotropy. If oxidated guanine directed DNA damage drives underlying age-related deterioration in cell function, it is expected that, likewise, there will be considerable stochastic mosaicism within aging tissues since not all cells are metabolically active in the same capacity. Thus, damaged cells are likely to closely coexist alongside relatively undamaged cells. One of the major unresolved issues concerns the frequency of seriously damaged cells necessary to reach a hypothetical precipice that kicks off a significant impairment of tissue function, which results in age-related diseases like AD and PD. Even more interesting, and needing further study, is whether there is a particular “fingerprint”, or key combination of signatures, in the type of damage and induced gene expression changes within vulnerable cells that later act as a catalyst for specific age-related diseases.
